# Diseases of the Stomach and Intestines

**Published:** 1904-05-14

**Authors:** 


### DISEASES OF THE STOMACH AND INTESTINES.

**Phlegmonous Gastritis.**—This interesting but rare condition which is known by a variety of names consists essentially of a suppurative inflammation of the gastric submucosa. Moynihan,[1] who has recently collected 81 cases from the literature adopts the usual classification into diffuse and circumscribed forms comparable to cellulitis and subcutaneous abscess respectively. The condition is about four times commoner in men than in women and has no marked age incidence. Primary cases are those originating in ulcer, carcinoma, or traumatism; secondary, those occurring in the course of acute infections. The symptoms in the diffuse form are not distinctive though very severe. A second stage, that of general peritonitis, is described, but this does not always supervene. The condition is invariably fatal and a positive diagnosis impossible. In the circumscribed form the symptoms are similar, but less severe and sometimes protracted. Recovery may be possible in a few cases. Rupture of the abscess into peritoneum or stomach may occur. In the latter event pus is vomited which does not occur in the diffuse form. This, or the discovery of a palpable tumour, does not distinguish the condition from perigastric abscess. Surgical treatment might be adopted in the early stages.

**Hour-glass Stomach.**—This is another rare condition of which an instance where frozen sections were made has recently been recorded by Dwight.[2] From these sections he concludes that during life the lesser curvature of the stomach is essentially vertical, and its main axis when empty almost horizontal from behind forwards. The causation of hour-glass contraction is occasionally found in ulcers or perigastric adhesions, but the main cause is, he thinks, the "fixation" of a normal contraction of the muscular fibres, only rarely congenital, and not due to any reversionary process.

**Diagnostic Methods.**—Two important points in the diagnosis of gastric disorders are the estimation of HCl and the detection of blood. The combined

HCl is frequently forgotten in this connection, but Fischer[3] proposes a method by which this may be estimated clinically with ease and rapidity. The free HCl is determined first with dimethyl-amidoazobenzol, and the total acidity with phenol-phthalein. The HCl is then exactly neutralised, the proteids precipitated with calcium phospho-tungstate, and the resulting filtrate decolorised with animal charcoal. Rosolic acid is then used to estimate the free HCl in this, and subtracted from the total acidity, the difference showing the amount of combined acid. Blood in small quantities can only be accurately determined by testing the fæces, the spectroscopic tests and the hæmin test in vomit being, according to Schmilinsky,[4] unreliable. Boas[5] recommends a modification of Weber's method, using only 5 to 10 grams of fæces. These are extracted with ether, treated with glacial acetic acid, and again extracted; the presence of blood in the extract is shown by a violet colour on the addition of gum guiacum and resinified turpentine oil. Schmilinsky states that minute quantities of blood only give a deep green or brownish-red tint. Bleeding from the intestine or anus must be excluded in gastric cases.

**Gastric Ulcer.**—The diagnosis of perforation in this condition is often difficult and sometimes impossible, especially in the early stages, when it is of the highest importance as an indication for immediate operation. This is illustrated by two cases reported by Peake.[6] In the first the patient had a good pulse, clean tongue, normal temperature and well-marked liver dulness an hour and a-half after perforation occurred, though subsequently death occurred from peritonitis. In the second, the patient, though not actually collapsed, had great abdominal pain and tenderness with little respiratory movement, a temperature of 102·4° and a pulse of 104. The liver dulness was absent. The subsequent course, however, showed that perforation had not occurred. In the treatment of gastric ulcer, discussed at the last meeting of the British Medical Association,[7] the value of withholding all food by the mouth was generally admitted. Nutrient enemata were usually recommended; Rolleston and Collier consider them merely *placebos*, but Boyd says they supply 210 calories daily. Ewart has devised a method for continuous rectal feeding. Dawson thinks they should not be continued for more than a week, but Griffiths allows them for 10 days. The administration of even small quantities of water by the mouth is condemned by Griffiths, Rolleston, Boyd, and Collier in the acute stage; Saundby allows milk and lime-water unless there is hæmatemesis, and King gives water in all cases. All agree that large amounts of water should be given *per rectum*, and that the bowel should be washed out—either daily (Rolleston) or every third day (Griffiths). As symptomatic treatment Rolleston advises bismuth, opium, silver nitrate, and counter-irritation. He lays much stress on the importance of oral asepsis; pyorrhœa alveolaris is common and causes much gastric irritation by the swallowing of septic matter. As curative treatment Rolleston and King advise iron in chlorotic cases; many chronic cases in men are syphilitic and should be treated by potassium iodide. Cautious lavage may be tried, or Einhorn's intragastric spray. Pyloric obstruction, recurrent hæmorrhage, or obstinate pain seem to indicate operation (Rolleston); but operation for hæmatemesis is of doubtful value (King, Boyd, Dawson, Collier). Rolleston considers ergot, adrenalin, lead, etc., as irrational drugs in hæmatemesis, but advises calcium chloride and citric acid if coagulability is impaired. Dawson and Parker also advocate the former drug in full doses. As bearing on the results of medical treatment Russell[8] has followed up the history of 47 cases of gastric ulcer in women for periods varying from 2 to 13 years after the first hæmatemesis. He finds that 2·1 per cent. died of the disease, 42·6 per cent. may be considered cured, 27·7 per cent. of these having had but one attack, 44·7 per cent. still suffered from gastric symptoms, and 6·4 per cent. remained in a doubtful condition. He considers that perhaps operations should be more frequently resorted to in chronic cases.

**Duodenal Ulcer.**—The occurrence of these ulcers after extensive burns has long been known; and in other cutaneous lesions they have also been occasionally observed. Borland[9] has recorded what he considers a unique case of duodenal ulcer which ended fatally by perforation, occurring in an infant suffering from eczema. When the latter disease became complicated by pyogenic infection the first symptoms of the ulcer appeared, and he regards the latter as caused by irritation of some part of the central nervous system by the absorbed toxins from the skin.

**Appendicitis.**—The history of the modern conception of appendicitis though short is of great interest, especially as British investigators were among the first to call attention to the condition. Kelly[10] attributes the first account of the disease to Parkinson (1812) and the first successful operation to Hancock (1848). Parker's publication in 1866 was the first, however, that attracted general attention. The morphology of the appendix, also an interesting study, has been described by Woods-Hutchinson.[11] It is a vestigial structure, useless to carnivorous animals and representing the third part of the cæcum. Martin,[12] in connection with the causation of appendicitis notes its occasional production by various intestinal worms. Boije[13] has collected a number of cases connected with the puerperium, but traces no causal relationship. Appendicitis is a serious complication of pregnancy, though pregnancy has little exacerbating effect on appendicitis. The treatment of appendicitis was discussed at the last British Medical Association meeting. Operation (apart from cases where pus was present) was generally thought by the physicians to be too frequently resorted to at the present day. King, in cases with pain and vomiting, when the pulse is 100 and the temperature 102°, gives nothing but a little water by the mouth, with full doses of opium and belladonna. Savil deprecates opium in such cases as masking symptoms, and rather inclines to early operation. Collier notes frequent recovery under merely medical treatment and the possibility of hernia or neuralgia in the site of an operation wound. In relapsing cases Odell advises operation after the second and Saundby after the third attack, but Tyson considers this premature interference. McPhedran states that in Canada early operation is the rule; both he and Saundby drew attention to the importance of the leucocyte count in diagnosing pus. In cases originating in colitis Saundby advises bowel irrigation with weak chinosol or nitrate of silver. Foster has observed the connection of the two conditions in four out of 30 cases, though Dieulafoy regards it as rare.

[1] Med. Chron., Nov., 1903, p. 76. [2] Am. Jour. Med. Sci., Oct., 1903, p. 581. [3] Ibid., p. 666. [4] Med. Rec., Jan. 2, 1904, p. 31. [5] Ibid., Dec. 12, 1903, p. 950. [6] Brit. Med. Jour., Nov. 21, 1903, p. 1327. [7] Ibid., Oct. 24, 1903, p. 1041 sqq. [8] Lancet, Jan. 30, 1904, p. 288. [9] Ibid., Oct. 17, 1903, p. 1084. [10] Brit. Med. Jour., Sept. 26, 1903, p. 749. [11] Ibid. [12] Ibid., Nov. 21, 1903, p. 1355. [13] Ibid. [14] Ibid., Oct. 24, 1903, p. 1034.

(*To be continued.*)

